# New Drugs for Pediatric Asthma

**DOI:** 10.3389/fped.2018.00432

**Published:** 2019-01-16

**Authors:** Marco Maglione, Marco Poeta, Francesca Santamaria

**Affiliations:** ^1^Department of Translational Medical Sciences, Federico II University, Naples, Italy; ^2^Department of Pediatrics, Federico II University, Naples, Italy

**Keywords:** severe asthma, therapy, biologics, inhaled corticosteroids, muscarinic antagonists, macrolides, children, adolescents

## Abstract

Asthma is the most common chronic disease in children. As suggested by international guidelines, the main goals of asthma treatment are symptoms control and lung function preservation, through a stepwise and control-based approach. The first line therapy based on inhaled corticosteroids may fail to reach control in more than one third of patients, especially adolescents, and in these lung function and quality of life may progressively worsen. Treatment with omalizumab, the first anti-immunoglobulin E recombinant humanized monoclonal antibody, has been definitely approved in pediatric uncontrolled asthma. In this review, we discuss the mechanisms and potential roles of emerging therapies for pediatric severe asthma. Novel biologic drugs (i.e., dupilumab, mepolizumab, reslizumab, and benralizumab) seem to be promising in reducing annual exacerbation rates and steroid-use in glucocorticoid-dependent cases, but available data are few and limited to adolescents and adults. Evidences on the use of the muscarinic antagonist tiotropium as controller medication in pediatric settings are progressively growing, sustaining an application as asthma maintenance treatment in children aged >6 years and in preschool children with persistent asthmatic symptoms, but well powered trials are needed to confirm its safety and efficacy. New inhaled corticosteroids (i.e., ciclesonide and mometasone) are effective as once-daily controller therapy, but long-term studies in the different pediatric ages are needed to compare effectiveness and safety to usual treatments. At present, the role of macrolides in pediatric severe asthma is controversial and their administration is not recommended routinely, but may be considered in children with neutrophilic asthma for reducing daily oral steroids administration and improving lung function. Despite the availability of several novel therapeutic strategies for uncontrolled asthma, future trials targeted at specific pediatric age subgroups are needed to support evidences of safety and efficacy also in children.

## Introduction

Asthma is the most common chronic condition of childhood, and its management represents part of the daily activity of most professionals who deal with pediatric care. At any age, the main purposes of asthma treatment are to reduce exacerbations and to limit the progressive loss of lung function, thus decreasing the use of health resources and improving quality of life. In few words, asthma therapy aims to achieve good symptom control (1).

National and international guidelines on asthma management of adults and children agree in indicating inhaled corticosteroids (ICS) as the most effective and safe medications, which may be used alone or associated with other controller therapies, in a stepwise, control-based approach (2–4).

Nevertheless, despite this approach, in more than one third of all patients with asthma the persistence of clinical symptoms, often associated with overt lung function abnormalities, indicate poor control, with the proportion increasing to >50% in adolescents (5, 6). This variability in treatment response is partly genetically determined. Pharmacogenomic studies, through the characterization of genes relevant in asthma treatment response, are paving the way toward the personalization of therapy. This would entail a dramatic change in asthma treatment from the actual “one size fits all” approach to the so called “precision medicine,” that is the tailoring of healthcare to the individual through the identification of clinical, biological, or genetic markers (7). Therefore, the interest in the development of new drugs and in the application of therapies currently used in other conditions for pursuing asthma control is high. This is particularly true in the pediatric setting, due to the lack of adequately designed trials including children. Indeed, even though results from adult studies may be sometimes translated to adolescents, this is not the case for younger asthmatic patients for whom evidence on the safety and efficacy of new treatments is still sparse.

The present review will go through the new therapeutic approaches to pediatric asthma, highlighting available evidence on their efficacy, the related risks, and the areas of uncertainty that in many cases still limit their regular application in the clinical practice.

## Biologics

Over the last decades, the development of monoclonal antibodies specifically designed to bind determined targets has deeply changed the approach to a number of conditions, also affecting children (8). In pediatric respiratory medicine, this novelty has been embodied by omalizumab, a recombinant DNA-derived humanized anti-IgE monoclonal antibody, which is the only biologic drug recommended in children with moderate-to-severe asthma (3, 9). Omalizumab, which was approved by the United States (US) and the European Union in 2003 and 2005, respectively, is able to decrease the quantity of cell-bound IgE, to downregulate the IgE receptors on mast cells, basophils, and dendritic cells, thus preventing mediator release from effector cells (10, 11). At present, its use is licensed as an add-on treatment for patients aged > 6 years with severe persistent allergic asthma and positive skin test or specific IgE to perennial aeroallergens, FEV_1_ < 80% predicted, frequent daytime symptoms, or nighttime awakenings, and multiple severe asthma exacerbations despite traditional maintenance therapy (12, 13). In comparison to the robust body of literature supporting the efficacy and safety of omalizumab in severe, inadequately controlled adult asthma (14, 15), evidence in children is far more limited. Nevertheless, even though no studies have been published to date in preschoolers, only few well-designed clinical trials have addressed the role of omalizumab in uncontrolled asthma affecting children older than 6 years. A large, multicenter randomized controlled trial (RCT) assessed the efficacy of omalizumab in 419 subjects (mean age 10.9 years) with persistent allergic asthma, and showed that anti-IgE treatment increases the number of symptoms-free days and reduces the number of exacerbations and the need for controller therapies (16). Similar results were achieved by other pediatric studies which have strengthened the role of omalizumab in limiting asthma exacerbations, and to a lesser extent, in improving patients' lung function (17–21). Despite reported cases of anaphylaxis in children (22), which warrant hospital administration, omalizumab is basically safe, whereas high cost represents a more relevant issue.

The scenario of biologics applied to asthma treatment has recently added to the more “consolidated” omalizumab, a number of novel drugs for which evidence of safety and efficacy is still very sparse and generally limited to adult studies. Dupilumab, an anti–interleukin-4 receptor α monoclonal antibody blocking both interleukin-4 and interleukin-13 signaling, is probably the most promising for a future application in pediatric asthma, as shown by two recent trials (23, 24). These large, multicenter RCT have enrolled patients aged >12 years with moderate-to-severe uncontrolled asthma (23) or glucocorticoid-dependent severe asthma (24). In such subjects dupilumab has proven effective in decreasing exacerbations and improving asthma control, also resulting in better lung function. Similar results have been provided for mepolizumab, which has been recently approved for severe eosinophilic asthma in adults and adolescents. This anti-interleukin-5 antibody has been shown to reduce severe asthma exacerbations (25) and need for oral steroids (26), even though evidence for its use in children < 12 years is virtually absent. Mepolizumab is not the only anti-interleukin-5 antibody under investigation for improving asthma control. Reslizumab and benralizumab are anti-interleukin-5 antibodies whose efficacy has been evaluated by few recent studies including patients with asthma aged >12 years (27–31). Both treatments have proven safe and effective in improving asthma control and lung function in selected patients with severe uncontrolled asthma and high blood eosinophil count, but, again, available data are few and limited to adolescents and adults.

## Muscarinic Antagonists

The increased cholinergic tone typical of asthma makes muscarinic receptors an obvious target for therapeutic strategies aimed at reducing airway hyperresponsiveness. In children with severe asthma exacerbations, inhaled short-acting antimuscarinic agents, namely ipratropium, are a widely accepted therapeutic option whose efficacy on lung function is well documented (32). Used in addition to nebulized albuterol, ipratropium has proven effective in reducing the risk for hospital admission as well as in improving spirometry in children with asthma exacerbations (33). Less frequent is the use of muscarinic antagonists as controller therapy, even though tiotropium, the most widely used long-acting muscarinic antagonist is mentioned as a possible add-on therapeutic option in step 4 of GINA guidelines for children older than 12 years taking the combined treatment of ICS and long-acting beta^2^-agonists (LABA), but still reporting inadequate asthma control (3). Inhaled tiotropium (Spiriva Respimat®), first indicated in adult COPD treatment, was shown to improve pulmonary function and respiratory symptoms in adult moderate-to-severe asthma (34, 35). Nevertheless, when added to ICS, tiotropium appears to be slightly less effective in improving quality of life in comparison to the traditional combination ICS/LABA (36). Tiotropium was recently approved by the US Federal Drug Administration (FDA) as an asthma maintenance treatment in children aged >6 years (37), whereas in Europe its use is still limited to adults. However, available evidence in children and adolescents is progressively growing, thus making a future wider application in pediatric asthma likely. In particular, school-aged children with severe symptomatic asthma have shown improvement of lung function and good tolerability and safety when tiotropium was added to medium or high-doses of ICS (38, 39). Furthermore, positive trends in asthma control and FEV_1_ were also observed in adolescents with moderately severe asthma treated with tiotropium as an add-on drug to ICS and other controllers for 3 months (40, 41). Finally, a recent small RCT showed the potential to reduce asthma exacerbation risk in children aged 1–5 years with persistent asthmatic symptoms, with tolerability similar to that of placebo, although mean daytime asthma symptom scores were not significantly different between groups (42). Despite its debated role within the group of controller drugs in childhood asthma, tiotropium remains an attractive option, both for the possibility of once-daily administration and for its peculiar way of delivery. Indeed, the drug is administered by a device named Respimat® in form of a mist of extremely fine particles (diameter < 6 μm), whose main advantages are the lower speed of delivery, the limited pharyngeal deposition and the enhanced pulmonary deposition in comparison to the common metered dose inhalers. Additional well powered trials are needed to further assess the safety and efficacy of tiotropium especially in young children with uncontrolled asthma symptoms.

## New Inhaled Corticosteroids

Inhaled corticosteroids represent the cornerstone of asthma therapy at all ages (3). Despite the long and large experience with traditional molecules such as beclometasone, flunisolide, fluticasone, and budesonide, all with high safety and efficacy profiles, some new ICS have been recently approved, but only few of these are allowed for the pediatric use.

Ciclesonide, licensed from age 4 years in the US and from age 12 years in Europe, is a pro-drug activated by esterases within the lung to form the active compound (des-ciclesonide). The possibility of a single daily administration, and evidence supporting its efficacy in improving asthma control and in reducing airway inflammation make this steroid a valid option as asthma controller therapy (43). Nonetheless, given the lack of relevant differences in the efficacy of ciclesonide vs. fluticasone or budesonide, long-term superiority trials are needed to identify the usefulness and safety of ciclesonide compared to other ICS (44). Similarly, mometasone, which has the same FDA approval as ciclesonide and may be used down to the age of 4 years also in Europe, has proven effective as once-daily controller therapy in several pediatric studies (45), with evidence supporting a significant functional improvement in school-aged children with persistent asthma (46).

## Macrolide Antibiotics

Macrolides are widely used antibiotics with both antimicrobial and anti-inflammatory activities (47). Indeed, in addition to their well-known antibiotic effect, there is evidence that macrolides modulate the expression of cellular adhesion molecules, may attenuate inflammatory cell migration and inhibit the respiratory burst in polymorphonuclear cells. Furthermore, macrolides clearly affect neutrophil function, even though the exact mechanisms have not been elucidated (48). For these reasons, macrolides have been initially recommended in diffuse panbronchiolitis (49), cystic fibrosis (50), and non-cystic fibrosis bronchiectasis (51). As airways infection is a possible cause for asthma, macrolides have been supposed to be used as long-term treatment for improving the disease control and reducing the need for steroids (52). Actually, the early troleandomycin—no longer recommended because of adverse effects on liver function tests—was reported to work as “steroid-sparing” drug by reducing the catabolism of steroids, but indeed no steroid reduction was demonstrated (53). Most recently, clarithromycin was found to widely suppress severe, steroid-insensitive allergic airways disease in a mouse model through its anti-inflammatory effects on tumor necrosis factor-α/interleukin-17 immune responses that are largely independent of its antimicrobial effects (54). Indeed, macrolides may reduce airway inflammation either by acting on pro-inflammatory cytokines or by controlling intracellular infection which may trigger and maintain inflammation (55).

At present, the role of macrolides in severe asthma is controversial. In a large multicentre RCT azithromycin did not reduce the rate of severe exacerbations and lower respiratory tract infections in a population of severe asthma adults not including children or adolescents (56). Finally, a systematic review and meta-analysis did not show a benefit of macrolides over placebo on rates of exacerbations, quality of life or participants' need for rescue medications (57).

Looking specifically at the pediatric literature, *Chlamydia pneumoniae* and *Mycoplasma pneumoniae* have been suggested to play a role in the pathogenesis of severe asthma (58). Preschool-aged children with frequent, severe exacerbations may benefit from sporadic use of azithromycin (59). Regrettably, very few RCTs investigated the role of macrolides in school-age children or adolescents with asthma, and failed to demonstrate a beneficial effect probably because of the low power of the study (60–62). Actually, the possibility that macrolides work well in pediatric severe asthma is not definitely excluded, yet at present the evidence is quite poor. It should be also kept in mind that an inappropriate use of macrolides unavoidably results in antibiotic resistance that is a major worldwide concern, especially in the pediatric population where the respiratory infection rate is as high as in the elderly (63).

Although an official document and a recent pragmatic review do not recommend the routine use of macrolides for children with severe asthma (9, 64), they may be useful for reducing daily oral steroid administration and improving FEV_1_ (65), and, for this reason, can be proposed as *ex-juvantibus* trial in children with neutrophilic asthma (66). In conclusion, further well-designed and large RCTs are warranted before routine use of macrolides is recommended or definitely condemned in pediatric severe asthma.

A list of new medications for treating children with severe, uncontrolled asthma, also including the novel evidence for their use, is provided in Table 1.

**Table 1:** Summary of novel drugs for treatment of severe pediatric asthma.

**Drug**	**Mechanism of action**	**Novel evidence in children**
**BIOLOGICS**		
Dupilumab	Anti–IL-4 receptor α monoclonal antibody, blocks both IL-4 and IL-13 signaling.	Two trials also involving children >12 years. Decreases exacerbations and improves lung function in moderate-to-severe uncontrolled asthma (22). In glucocorticoid-dependent asthma reduces steroid use (23).
Mepolizumab	Anti-IL-5 monoclonal antibody, selectively inhibits eosinophilic inflammation.	Two trials also involving children >12 years. Effective and well tolerated. Reduces the risk of asthma exacerbations in patients with severe eosinophilic asthma (24, 25).
Reslizumab	Anti-IL-5 monoclonal antibody, inhibits activity within the IL-5 signaling pathway and reduces blood and tissue eosinophils.	Three trials also involving children >12 years (26, 27, 29). Improves lung function, asthma control and symptoms, quality of life in uncontrolled asthma.
Benralizumab	Anti-IL-5 receptor α monoclonal antibody, induces direct, rapid, and nearly complete depletion of eosinophils.	Two trials also involving children >12 years (28, 30). Significantly reduces annual exacerbation rates in patients with severe, uncontrolled asthma with blood eosinophils >300 cells per μL.
**MUSCARINIC ANTAGONISTS**		
Tiotropium	Long-acting muscarinic antagonist, decreases airway tone binding the muscarinic receptors.	Evidence of improved lung function in children >12 years receiving tiotropium in addition to standard therapy (39, 40), as well as in children aged 6–11 years (37, 38). Weaker evidence of reduction in exacerbations rate in children aged 1–5 years (41).
**NEW INHALED CORTICOSTEROIDS**		
Ciclesonide	Reduces airway inflammation through a single day administration	Improves airway inflammation and asthma control in atopic children (42). Longer-term trials needed to identify the usefulness and safety compared to other ICS (43).
Mometasone	Reduces airway inflammation through a single day administration	Functional improvement in school-aged children with persistent asthma (45).
**MACROLIDES**		
	Reduce airway inflammation by acting on pro-inflammatory cytokines or by controlling intracellular infection.	May reduce daily oral steroid administration and improve FEV_1_ (63). Routine use not recommended in children or adolescents with severe asthma (8, 62).

## Other Interventions

Immunosuppressive drugs including cyclosporine, azathioprine and methotrexate, commonly used in several immune-mediated disorders, have been proposed for severe asthma management, but no recommendation may be formulated at present due to the insufficiency of data, particularly in children (3).

Allergen immunotherapy has proven effective in improving symptom control in mild to moderate asthmatic children, especially in the presence of a clear association between symptoms and exposure to a specific allergen, but its applicability in the clinical practice is strongly limited by the requirement for patients to have stable (and not uncontrolled) asthma symptoms due to the risk of severe reaction (67).

A surgical procedure named bronchial thermoplasty, consisting in the ablation of the airway smooth muscle layer, has shown to provide some benefits in adults unresponsive to conventional therapies (68). Nevertheless, evidence in children or adolescents is completely lacking at present, and such intervention is therefore not recommended in these age groups.

Finally, the association between worse symptom control and fungal sensitization in severe asthma has led to studies assessing the efficacy of antifungal drugs on asthma outcomes. However, data are still conflicting and not conclusive and insufficient to support formal recommendations (69).

## Conclusions

Severe asthma identifies children or adolescents who need high-dose ICS therapy and a second controller therapy in the previous year, or systemic corticosteroids for 50% of the year, to prevent asthma from being uncontrolled or that remains uncontrolled notwithstanding these medications (9). Unfortunately, once excluded any comorbidity or optimized patients' adherence to treatment and inhalation technique, about 5–10% of the asthmatic pediatric population continue to have severe symptoms or signs and the loss of lung function may be progressive and irreversible (70). The latter point is of paramount importance in view of the fact that a stringent relationship between the childhood insults to the lung and the accelerated aging that can occur in adult chronic obstructive lung disease has been postulated (71). Finally, asthma not responding to treatment may result in significant morbidity and frequent healthcare utilization (1, 72). For all the above, there is considerable need for robust studies of children with uncontrolled asthma confirming the clinical efficacy and safety of medications increasingly used in the adult population, but not allowed in patients < 12 years of age because of the paucity of literature data.

## Author Contributions

MM has made substantial contributions to conception and design, has been involved in drafting the manuscript, and has given final approval of the version to be published. MP conceived the idea, has been involved in drafting the manuscript and has given final approval of the version to be published. FS has made substantial contributions to conception and design, has been involved in drafting the manuscript and revising it critically for important intellectual content, and has given final approval of the version to be published.

### Conflict of Interest Statement

The authors declare that the research was conducted in the absence of any commercial or financial relationships that could be construed as a potential conflict of interest.
